# Mitigating prolonged QT interval in cancer nanodrug development for accelerated clinical translation

**DOI:** 10.1186/1477-3155-11-40

**Published:** 2013-12-14

**Authors:** Amalendu P Ranjan, Anindita Mukerjee, Lawrence Helson, Jamboor K Vishwanatha

**Affiliations:** 1 Department of Molecular Biology & Immunology and Institute for Cancer Research, Graduate School of Biomedical Sciences, University of North Texas Health Science Center, Fort Worth, Texas 76107, USA; 2 SignPath Pharmaceuticals, Pennsylvania, USA

**Keywords:** QT prolongation, hERG, Curcumin, Nanoparticle, Hybrid

## Abstract

**Background:**

Cardiac toxicity is the foremost reason for drug discontinuation from development to clinical evaluation and post market surveillance [Fung 35:293-317, 2001; Piccini 158:317-326 2009]. The Food and Drug Administration (FDA) has rejected many potential pharmaceutical agents due to QT prolongation effects. Since drug development and FDA approval takes an enormous amount of time, money and effort with high failure rates, there is an increased focus on rescuing drugs that cause QT prolongation. If these otherwise safe and potent drugs were formulated in a unique way so as to mitigate the QT prolongation associated with them, these potent drugs may get FDA approval for clinical use. Rescuing these compounds not only benefit the patients who need them but also require much less time and money thus leading to faster clinical translation. In this study, we chose curcumin as our drug of choice since it has been shown to posses anti-tumor properties against various cancers with limited toxicity. The major limitations with this pharmacologically active drug are (a) its ability to prolong QT by inhibiting the hERG channel and (b) its low bioavailability. In our previous studies, we found that lipids have protective actions against hERG channel inhibition and therefore QT prolongation.

**Results:**

Results of the manual patch clamp assay of HEK 293 cells clearly illustrated that our hybrid nanocurcumin formulation prevented the curcumin induced inhibition of hERG K^+^ channel at concentrations higher than the therapeutic concentrations of curcumin. Comparing the percent inhibition, the hybrid nanocurcumin limited inhibition to 24.8% at a high curcumin equivalent concentration of 18 μM. Liposomal curcumin could only decrease this inhibition upto 30% only at lower curcumin concentration of 6 μM but not at 18 μM concentration.

**Conclusions:**

Here we show a curcumin encapsulated lipopolymeric hybrid nanoparticle formulation which could protect against QT prolongation and also render increased bioavailability and stability thereby overcoming the limitations associated with curcumin.

## Background

Cardiac safety is very critical for any drug development. Since drug development often takes many years and requires an investment of millions of dollars to achieve FDA approval, it becomes critical to determine the cardiac safety and translational possibilities early in the development process [1-5]. In the early 1990s, several marketed drugs were withdrawn due to their arrhythmogenic risk. On a standard ECG, these drugs prolonged the QT interval which is the duration measured from the beginning of the QRS complex (depolarization of the cardiac myocytes) to the end of the T wave (completion of the repolarization phase of the cardiac myocytes). A prolonged QT interval may potentially induce torsade des pointes (TdP) which is a form of ventricular arrhythmia [6]. Repolarization of the cardiac action potential is a result of currents generated by an outward flow of K^+^ through the K^+^ channels. Obstruction of ion flow in the channel leads to delayed repolarization as evidenced by a prolonged QT interval [7]. This led the International Conference on Harmonization of Technical Requirements for Registration of Pharmaceuticals for Human Use to issue the E17 guideline in 2005. This guideline was directed at the clinical evaluation of QT interval prolongation and proarrhythmic potential of non-antiarrhythmic drugs. During drug development and phase I-II studies, it is important to ascertain the potential of the drug to cause (a) direct myocyte toxicity, (b) changes in cardiac repolarization, (c) prolong QT interval, (d) effects on vascular tone and injury [8], or (e) induction of life threatening proarrhythmias. The guideline required that the effect on cardiac repolarization of every drug, including anticancer agents, should be evaluated before phase II trials [9].

Several drugs can interfere with hERG (human ether-a-go-go-related gene) coded potassium channels, which are responsible for the rapid component of repolarisation. This drug induced inhibition may ultimately lead to prolongation of the QT interval [10]. The hERG (human ether-a-go-go related gene) encodes for the α-subunit of the ion channel that is responsible for the rapidly activating delayed rectifier potassium channel I_Kr_ (K_v_11.1). The three-dimensional structure of the hERG channel is still unknown although site-directed mutagenesis data have provided some insight into its structure [11]. Within the hERG channel pore cavity, ion flux and currents can be modified depending upon the open or closed states, and by drug interactions at key high affinity drug binding sites. These sites are the aromatic amino-acid residues (Y652 and F656) on the inner helices of the pore. The most important currents mediated by drugs are the sensitive delayed I_Kr_ (rapid) current which repolarizes the myocardial cells and the I_Ks_ (slow) rectifier currents are exhibited on the standard ECG as the QT interval. The corrected current with heart rate is defined as QTc [7]. This I_Kr_ or the hERG current is critical for ventricular repolarization and inhibition of I_Kr_ can induce QT prolongation which may result in life threatening ventricular arrhythmias [12].

Curcumin is a drug molecule that has an enormous clinical potential as a potent anti-cancer, anti-oxidant, anti-inflammatory and anti-bacterial drug in addition to its many other pharmacological properties. Unfortunately, curcumin has been reported to block the hERG K^+^ potassium channel [7,13]. Studies revealed that curcumin inhibited the hERG K^+^ channel in a concentration dependent manner with an IC_50_ as low as 5.5 μM. A low concentration of 11.4 μM of curcumin resulted in an 80% inhibition of hERG tail current density leading to prolongation in the QT interval [13]. Since curcumin induces QT prolongation, it is unsafe for clinical translation unless there are ways to inhibit its QT prolonging properties. Until now there has been only one study on QT prolongation caused by curcumin. This particular study shows that liposomal formulation of curcumin is able to prevent QTc prolongation [7]. However, the concentration of liposomal curcumin which is able to provide this protection is still much lower than the therapeutic concentration.

**Figure 1:**
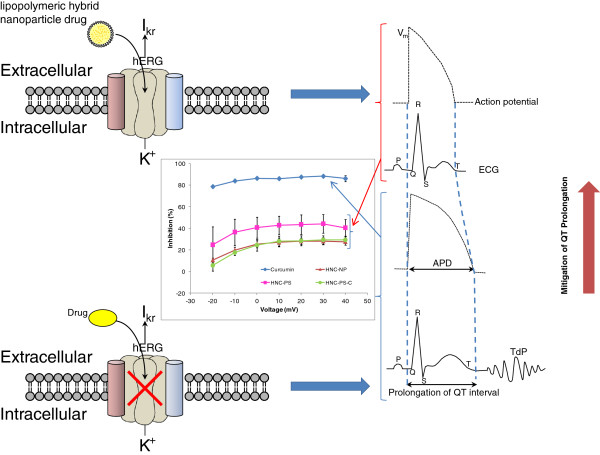
Schematic diagram depicting the mitigating of prolonged QT interval using nanodrug.

In the present study, we developed a nanoformulation of curcumin that may mitigate hERG channel current inhibition by encapsulating therapeutic concentrations of curcumin within lipopolymeric hybrid nanoparticles in order to block the inhibition of hERG K^+^ channels. A representation of the same is illustrated in Figure 1. We believe this would result in a safer clinical product and FDA approval. Compared to other curcumin nanoformulations, our nanocurcumin formulation provides improved bioavailability and better stability which are critical for successful scalability and long shelf life. Using stably transfected Human Embryonic Kidney cells (HEK 293 cells), this study tested the *in vitro* effects of three different lipopolymeric hybrid nanocurcumin formulations in comparison to liposomal curcumin and curcumin on the potassium-selective I_Kr_ current generated in normoxic conditions.

## Materials and methods

### Materials

E-4031, a class III anti-arrhythmic drug is a synthetic toxin used solely for research with one clinical exception [14]. Its mechanism of action is to block the hERG voltage-gated K^+^ channels. E-4031, purchased from Sigma-Aldrich, was selected as a positive control compound for this study. Three naive HEK293-hERG cells were exposed to 100 nM E-4031.

Curcumin, 99.2% pure, was synthesized under GMP conditions by Sabinsa (NJ, USA) and obtained through SignPath Pharma Inc. The GMP grade liposomal curcumin was obtained from Polymun GmbH (Vienna, Austria). The liposomal curcumin were made with a 9:1 ratio of DMPC (1,2-dimyristoil-*sn-*glycero-3-phosphocholine) and DMPG (1,2-dimyristoyl-*sn*-glycero-3-phospho-rac-[1-glycerol]) with a curcumin content of 6.4 mg/ml.

The concentration of various curcumin nanoformulations and curcumin (6, 12 and 18 μM) were selected based on predicted human plasma levels. The selected concentrations reflected a range estimated to exceed the therapeutic doses and provide valuable predictions of the effect of curcumin and various nanocurcumin formulations on human cardiac electrophysiology.

### Formulation of lipopolymeric hybrid curcumin nanoparticles

Three different formulations of curcumin encapsulated lipopolymeric hybrid nanoparticles were prepared to identify the best suited formulation.

### Formulation A (HNC-NP): nanoprecipitation

Curcumin was dispersed in an organic phase containing PLGA in acetonitrile. The lipids, DMPC (1,2-dimyristoil-*sn-*glycero-3-phosphocholine) and DMPG (1,2-dimyristoyl-*sn*-glycero-3-phospho-rac-[1-glycerol]) were used at a pre-determined ratio (7:3). There are previous reports of liposomes composed of the same lipids (i.e. DMPC and DMPG), which lacked general toxicity and decreased nystatin toxicity [15]. Hence we chose to use the same combination of lipids but optimized the ratio in a previous study to obtain the optimal formulation. DMPG was dissolved in double distilled water while DMPC was dissolved in 4% ethanol water. Both lipids were then mixed and heated to 45°C. The organic phase mixture was subsequently added dropwise with constant stirring. The mixture was stirred for 3 hours on magnetic stirrers to facilitate evaporation of the organic solvent from the formulation. Once the organic solvent was evaporated, the mixture was centrifuged thrice at 6000 rpm for 15 minutes using Amicon filters with a 10 kd cutoff. The nanoparticles so obtained were resuspended in a 5% sucrose that acts as a cryoprotectant. Further, the nanoparticle-cryoprotectant mixture is flash frozen in liquid nitrogen for 5 minutes and then lyophilized overnight.

### Formulation B (HNC-PS): probe sonication

Curcumin was dispersed in an organic phase containing PLGA in ethyl acetate. The lipids, DMPC (1,2-dimyristoil-*sn-*glycero-3-phosphocholine) and DMPG (1,2-dimyristoyl-*sn*-glycero-3-phospho-rac-[1-glycerol]) were used in the same ratio (7:3). DMPG was dissolved in double distilled water while DMPC was dissolved in 4% ethanol water. The lipid mixture and the PLGA-curcumin solution were mixed and sonicated for 1 minute. Subsequently, the nanoparticles were centrifuged at 6000 rpm for 15 minutes using Amicon filters with a 10kd cutoff and then washed thrice. The nanoparticles were resuspended in a 5% sucrose, flash frozen in liquid nitrogen and lyophilized overnight.

### Formulation C (HNC-PS-C): probe sonication with chitosan blended polymer

In this protocol, chitosan was added during the formulation of curcumin loaded PLGA nanoparticles using ethyl acetate by solvent evaporation in order to provide a positive surface charge to the nanoparticles [16]. Since lipids mostly carry negative charge, this method leads to a tighter lipid layer on the surface of the nanoparticles. The formulation was carried out as described under probe sonication.

### Characterization of lipopolymeric hybrid curcumin nanoparticles

The lipopolymeric hybrid nanocurcumin from all three batches were characterized for their drug loading as previously described. Particle size and zeta potential are important parameters as they have an effect on both the intracellular uptake as well as the stability of the nanoparticles. Average particle sizes of the nanoparticles were determined by Nanotrac Particle Size Analyzer (Mircotrac, Inc., Montgomeryville, PA). Nanoparticles were characterized for zeta (ζ) potential by using a Zetasizer (ZEN4602, Malvern Instruments, Worchestershire, UK). The dried nanoparticles were suspended in distilled Milli Q treated water (pH 7) and measured. Zeta potential was calculated based on electrophoretic mobilityof the nanoparticles in aqueous medium. Surface morphology was determined by Transmission Electron Microscopy (TEM) as described elsewhere [17]. Further, stability analysis was also carried out at 4°C for all the three batches of nanoparticles formulated.

### Determination of hERG current inhibition

The compounds tested for hERG current inhibition include lipopolymeric hybrid nanocurcumin (HNC-NP, HNC-PS, HNC-PS-C), liposomal curcumin and curcumin. E-4031 was selected as the positive control to confirm the sensitivity of the test system. This study was carried out at IPS Therapeutique, Inc., QC, Canada.

### Manual patch clamp assay

Whole cell patch-clamp recordings were made at 35 ± 2°C from HEK 293 cells stably transfected with the hERG gene. Cells were plated onto 35 mm petri dishes (Falcon) and maintained in culture with the selective agent G148 (Sigma-Aldrich, St. Louis). Cells were washed twice with 1 mL of hERG external solution containing (mM) NaCl 140, KCl 5, CaCl_2_ 1.8, MgCl_2_ 1, Glucose 10 and HEPES 10 (pH 7.4) followed by the addition of 2 mL of hERG external solution. The petri dish (experimental chamber) was mounted on the stage of an inverted phase contrast microscope. A borosilicate glass micropipette (open resistance of 5 to 15 MΩ) pulled with a PMP-102 Programmable Micropipette Puller and filled with internal pipette solution containing (in mM) KCl 140, MgCl_2_ 1, EGTA 5, HEPES 10, Mg-ATP 4 and sucrose 10 was positioned above a single cell using an Eppendorf PatchMan micromanipulator. The micropipette was lowered to the cell until a close contact was achieved. The whole-cell configuration was then obtained by applying a slight negative pressure (resistances were measured by a 5-mV square pulse to be in the GΩ range). Cell capacitance was immediately measured in order to evaluate cell surface area, using a conversion factor of 1 pF/m^2^. This cell surface area was later used to calculate net current density (as opposed to current amplitude).

Currents were acquired at a rate of 1 kHz, and filtered using a low-pass 4-pole Bessel filter with the cut-off rate set at 500 Hz. Baseline condition currents were recorded using the PClamp 10 acquisition suite (Molecular Devices, Sunnyvale, CA) following a 2-minute equilibration period. Currents were then recorded after 5 minutes of exposure to each concentration of test compounds or positive controls (lipopolymeric hybrid nanocurcumin, liposomal curcumin, curcumin, E-4031). The cells were stimulated continuously with the following pulse:

Pulses Protocol

Currents were analyzed using the Clampfit 10.2.0.14 module of the pClamp 10.2.0.14 software (Axon Instrument Inc., Foster City, California, USA, (now Molecular Devices Inc.).

### Current run-down and solvent effect correction

All data points have been corrected for solvent effect and time-dependent current run-down. Current run-down and solvent effects were measured simultaneously by applying the experimental design in test-compound free conditions (dimethyl sulfoxide vehicle) over the same time frame as was done with the test compounds. The loss in current amplitude measured during these vehicle control experiments (representing both solvent effects and time-dependent run-down) was subtracted from the loss of amplitude measured in the presence of the test compound. This procedure isolates the effect of the test compound from the effect of the solvent and the inevitable run-down in current amplitude over time.

### Application of test compounds, positive control and vehicle

Cells were exposed for 5 minutes to each concentration of compounds in the presence of closed circuit perfusion (2 mL/min). The cells were allowed 5 minutes for washout in the presence of a flow-through perfusion (2 mL/min) in addition to a closed circuit perfusion (2 mL/min). The positive control (100 nM; E-4031) was added to naive cells obtained from the same cell line and passage number for a period of 5 minutes in presence of a closed circuit perfusion (2 mL/min). Cells were under continuous stimulation of the pulses protocol throughout the experiments and cell currents were recorded after 5 minutes of exposure to each condition.

### Statistical analysis

Statistical comparisons were made using paired Student’s t-tests. For the test compounds, the currents recorded after exposure to the different test compound concentrations were statistically compared to the currents recorded in baseline conditions. Currents recorded after the washouts were compared to the currents measured after the highest concentration of test compounds. In the same way, currents recorded after the positive control was compared to the currents recorded in baseline conditions. Differences were considered significant when p ≤0.05.

## Results and discussion

### Formulation and characterization of lipopolymeric hybrid curcumin nanoparticles

**Figure 2:**
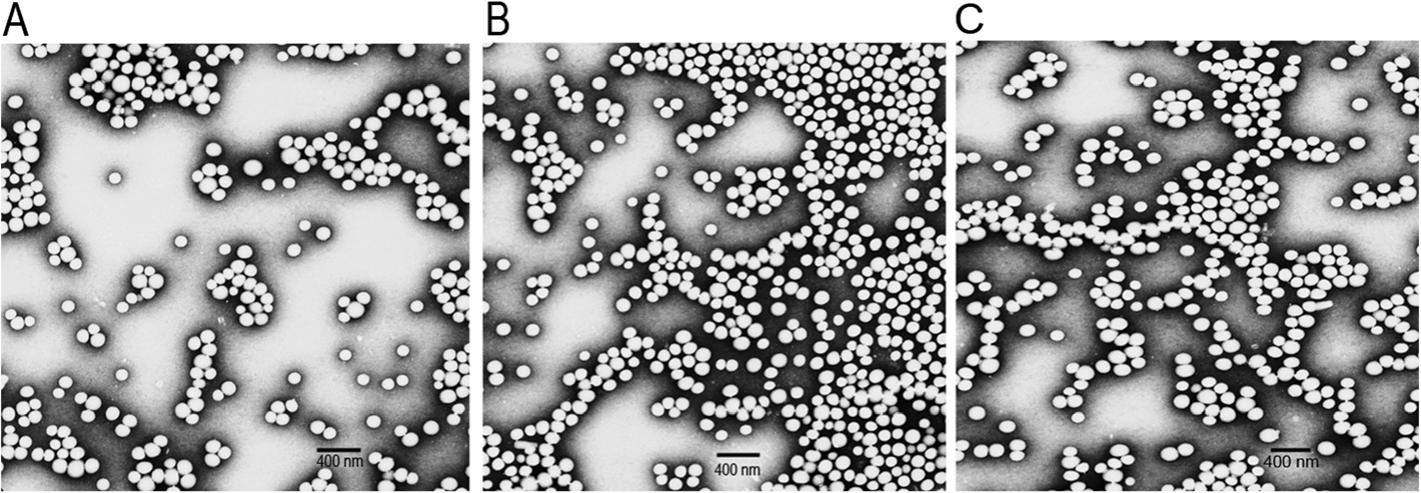
Transmission electron microscopy: A) HNC-NP; B) HNC-PS and C) HNC-PS-C formulations showing smooth and spherical monodispersed nanoparticles.

**Figure 3:**
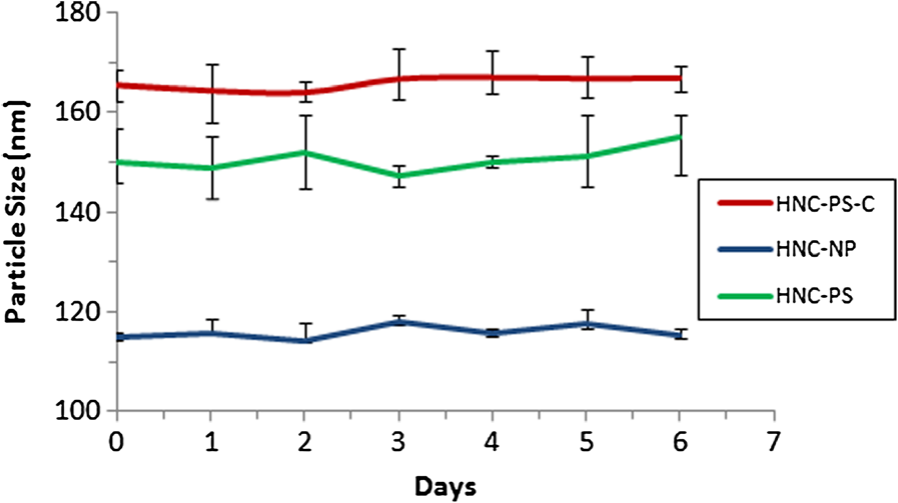
**Stability analysis of hybrid nanoparticles at 4°C for 6 days.** No significant change in particle size is observed for any batch.

Our results reveal that all three different formulations of the curcumin encapsulated lipopolymeric hybrid nanoparticles (HNC-NP, HNC-PS and HNC-PS-C) were successful. The first formulation (e.g. HNC-NP) was prepared by nanoprecipitation. The other two formulations (e.g. HNC-PS and HNC-PS-C) were prepared by probe sonication with or without chitosan. The hybrid nanoparticles were characterized for curcumin content or drug loading. We observed that the HNC-NP and HNC-PS-C formulations had a curcumin loading of 18 ± 0.8 μg/mg and 18.2 ± 1.2 μg/mg respectively. The HNC-PS formulation had a lower curcumin content of 6.8 ± 0.74 μg/mg. The particle sizes of the nanoparticles for the different batches were 113.9 ± 8.3, 147.2 ± 15.7, 162.2 ± 12.4 nm respectively. The zeta potential of the batches was found to be -24.5 ± 1.7 mV for HNC-NP, -24.8 ± 2.9 mV for HNC-PS and -21.1 ± 2.3 mV for HNC-PS-C. The slight change in the HNC-PS-C can be explained by the incorporation of chitosan which may impart some positive charge. The transmission electron microscopy (TEM) images show the surface morphology of the hybrid nanoparticles to be smooth and spherical (Figure 2A-C). The stability analysis was carried out for all the three batches of nanoparticles formulated at 4°C for 6 days. Results (Figure 3) show no significant change in particle size of the nanoparticles for the entire duration of the study ensuring that the hybrid nanoparticle are stable for atleast 6 days at 4°C.

### Effect of different formulations on whole-cell I_Kr_ hERG currents

The hERG assay or the Manual Patch Clamp assay is used to assess the potential of a compound to interfere with the rapidly activating delayed-rectifier potassium channel. This inital study used stably transfected Human Embryonic Kidney cells (HEK 293 cells) in normoxic conditions to quantify the *in vitro* effects of lipopolymeric hybrid nanocurcumin formulations, (HNC-NP, HNC-PS and HNC-PS-C), liposomal curcumin and free curcumin on the potassium-selective I_Kr_ current. The study design was based on the current International Conference on Harmonisation (ICH) Harmonized Tripartite Guidelines [9].

**Figure 4:**
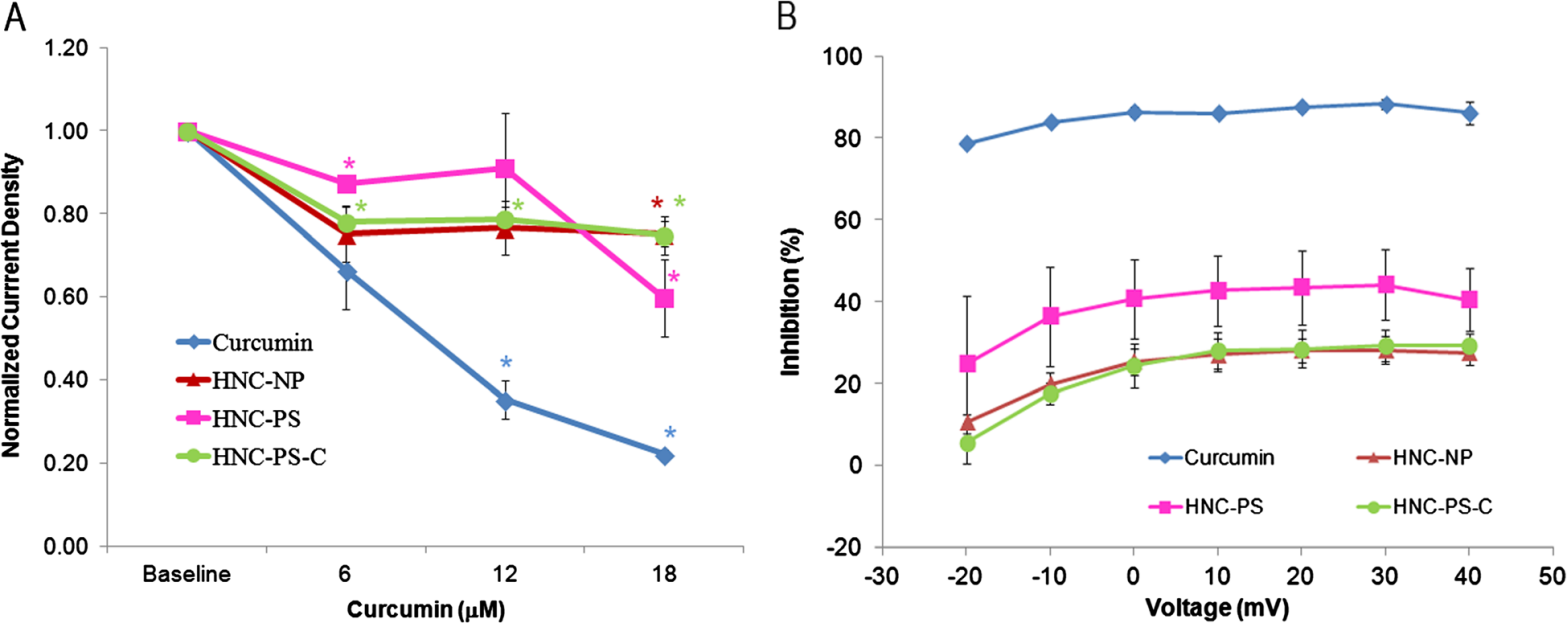
**hERG tail current density averages obtained by measuring the hERG tail peak amplitude at 20 mV in baseline conditions and in the presence of curcumin, Formulation HNC-NP, Formulation HNC-PS and Formulation HNC-PS-C hybrid nanocurcumin.** The curcumin concentrations were always 6.0, 12 and 18 μM. **A**: Current density was measured from 7 cells, averaged, normalized against baseline current density, and corrected for time and solvent effects. Statistical comparisons between post-drug exposure and baseline current density levels were made using repeat paired Student’s t-tests (*). Differences were considered significant when p ≤ 0.05. **B**: Voltage dependency of the hERG tail currents inhibition at the higher concentration of curcumin and hybrid nanoformulations tested (18 μM).

Whole-cell currents elicited during a voltage pulse were recorded in baseline conditions and following the application of the selected concentrations of test compounds. Currents were also recorded following a washout period. The cells were depolarized for one second from the holding potential (-80 mV) to a maximum value of +40 mV, starting at -40 mV and progressing in 10 mV increments. The membrane potential was then repolarized to -55 mV for one second, and finally returned to -80 mV. Whole-cell tail current amplitude was measured at a holding potential of -55 mV, following activation of the current from -40 to +40 mV. Current amplitude was measured at the maximum (peak) of this tail current. Current density was obtained by dividing current amplitude by cell capacitance measured prior to capacitive transient minimization. Each of the test compounds was analyzed at three different concentrations (6, 12 and 18 μM) for hERG current inhibition. The effect of test compounds on hERG current density is shown in Table 1. The results of the Manual Patch Clamp assay reveal that lipopolymeric hybrid nanocurcumin formulations were able to protect against the inhibition of hERG current caused by curcumin. Out of the three hybrid nanocurcumin formulations, formulation HNC-NP was found to impart the best protection as seen in Figure 4. At a concentration of 18 μM of curcumin equivalent, the HNC-NP formulation caused the least inhibition of current density (24.8% inhibition) followed by HNC-PS-C (25.2% inhibition) when compared to 77.8% inhibition caused by curcumin alone. Paired student’s t-tests confirmed that the difference in normalized current density measured at baseline and in the presence of 18 μM of lipopolymeric hybrid nanocurcumin reached the selected threshold for statistical significance (p ≤ 0.05). The liposomal curcumin formulation, at a curcumin concentration of 6 μM, induced a statistically significant inhibition of the hERG tail current (30%) at I_+20_ (n = 3) (not included in figure). E-4031 (a positive control) induced a significant inhibition of the current amplitude (81.8%) for I_+20_ at a concentration of 100 nM. Additionally, poly (lactic-coglycolic acid) (PLGA), at concentrations ranging from 0.52 to 29.81 μg/mL did not cause a statistically significant inhibition of the hERG tail current density for I_+20_ (n = 3). This result implies that formulation HNC-NP reduced the hERG current inhibition by 68.1% at a concentration as high as 18 μM which represents a significant protection of the hERG channel current inhibition caused by curcumin. To confirm the reversal effect of the test compounds, cells exposed to the highest concentration of curcumin equivalent formulation (18 μM) were subjected to a washout period of 5 minutes. The current measured after the washout period was not statistically different when compared to the current following exposure to the highest concentration of the compounds suggesting that the effect of these compounds was not reversible at this time point.

**Table 1:** **Effect of tested compounds on hERG current density at +20 mV (I**_**+20**_**)**

**Compound tested**	**Statistically significant inhibition starting at (μM)**	**Maximal inhibition (%)**	**Statistically significant reversibility of the Washout**	**Calculated IC50 (μM)**	**Voltage dependence**
**HNC-NP**	18	24.8	No	n/a	Yes
**HNC-PS**	6	40.2	No	n/a	Yes
**HNC-PS-C**	6	25.2	No	n/a	Yes
**Curcumin**	12	77.8	No	8.5	Yes
**Liposomal curcumin**	6 only	30	No	n/a	No

It has been previously reported that curcumin interacts with the alpha subunit of the K^+^ channel which forms the pore of the hERG channel [7]. It is this subunit which is encoded by the hERG gene. Maati et al reported a similar study showing that a curcuminoid mixture containing 78% curcumin brought about comparable inhibition of the hERG current [18]. A study by Helson et al demonstrated that liposome may have a protective effect against the inhibitory interactions of active compounds with channel drug receptor sites allowing more normal gating kinetics to occur. However, they could not confirm whether the changes in gating kinetics were due to a direct interaction of curcumin with the pore-forming peptides, or whether a change in lipid packing in proximity of the hERG channel resulted in decreased inhibition [7]. However, the concentration of liposomal curcumin which is able to provide this protection was much lower than the therapeutic concentration of curcumin and the liposomal formulation was found to be stable only at -20°C [7].

The results of the present study (Table 1) confirm that the curcumin (diferuloylmethane) molecule exhibits prominent I_Kr_ inhibition as reported by other research groups [7,13]. In the present study, our formulation of lipopolymeric hybrid nanocurcumin formulation was conceptualized based on the protective effects of liposomes on curcumin dependent inhibition of the hERG current. Further, the introduction of a polymer layer in the hybrid formulation imparted much needed stability and tunability to the formulation. The manual patch clamp assay of HEK 293 cells clearly illustrated that this hybrid nanocurcumin formulation prevented the curcumin induced inhibition of hERG K^+^ channel at concentrations higher than the therapeutic concentrations of curcumin. Liposomal curcumin, on the other hand, could only decrease this inhibition at lower curcumin concentration (6 μM). Comparing the percent inhibition, the hybrid nanocurcumin limited inhibition to 24.8% at a high curcumin equivalent concentration of 18 μM (n = 3; statistically significant). This is substantially less than the inhibition (30%) brought about by liposomal curcumin at one third the concentration (6 μM curcumin equivalent). There remains a need to determine whether the changes in gating kinetics were brought about due to direct interaction of curcumin with pore-forming peptides, or whether a change in lipid packing in proximity of the hERG channel resulted in decreased inhibition. Moreover, the present formulation provided improved bioavailability [19] (data not shown) and as expected, this formulation was stable both at 4°C for an extended period of time.

## Conclusions

The inhibition of the I_Kr_ current induced by curcumin is mitigated when curcumin is incorporated within a lipopolymeric hybrid nanoparticle formulation prior to exposure. Such a formulation not only protects inhibition of hERG channel current induced by curcumin but also provides improved bioavailability and stability. Similar hybrid formulations may be used for other QT-prolonging drugs to mitigate prolonged QT effects *in vitro.* Further testing of the hybrid nanoformulation with other QT-prolonging drugs in animal models with QT-prolongation may lead to faster clinical translation and FDA approval of such cardiotoxic yet pharmacologically potent drugs.

## Competing interests

The authors declare that they have no competing interests.

## Authors’ contributions

APR and AM conceived the concept, designed the experiments, prepared nanoparticles, analyzed the experiments and wrote the paper. LH conceived the concept and reviewed the paper. JKV mentored, provided the resources, supervised the experiments and reviewed the paper. All authors read and approved the final manuscript.
